# Cultural translation of the constipation assessment a study on the reliability and validity of the Chinese version of the constipation severity index

**DOI:** 10.3389/fmed.2025.1602198

**Published:** 2025-06-23

**Authors:** Wei Hu, Madhulika G. Varma, Xiaoli Huang, Xin Wang, Ke Shang, Di Xu, Xia Li

**Affiliations:** ^1^First Affiliated Hospital of Jinzhou Medical University, Jinzhou, China; ^2^Department of Surgery, University of California, San Francisco, San Francisco, CA, United States; ^3^School of Nursing, Jiangxi Medical College, Shangrao, Jiangxi, China; ^4^Huaian Hospital of Huaian City, Huai'an, Jiangsu, China; ^5^School of Nursing, Jinzhou Medical University, Jinzhou, Liaoning, China

**Keywords:** constipation severity index, China, psychometric properties, validation, chronic constipation, patient-reported outcome measure

## Abstract

**Background:**

Assessing the severity of constipation in patients is important for tailoring treatment plans and monitoring outcomes. However, validated assessment tools for constipation severity are limited in China.

**Objective:**

This study aimed to evaluate the psychometric properties of the Chinese version of the Constipation Severity Index (CSI).

**Methods:**

A cross-sectional survey was conducted in two tertiary hospitals in China. A total of 621 patients meeting the diagnostic criteria for constipation were enrolled. The scale’s reliability and validity were assessed using Content Validity Index (CVI), Exploratory Factor Analysis (EFA), Confirmatory Factor Analysis (CFA), internal consistency, and test–retest reliability.

**Results:**

The expert-rated Item-level Content Validity Index (I-CVI) was 0.90. EFA revealed a three-factor structure comprising 16 items, accounting for 71.81% of the total variance. CFA results suggested acceptable model fit (χ2 = 257.711, df = 96, *p* < 0.001; CFI = 0.965; TLI = 0.956; RMSEA = 0.074, 90% CI: 0.063–0.085; SRMR = 0.047). The scale demonstrated good internal consistency (Cronbach’s alpha = 0.936, McDonald’s Omega = 0.937).

**Conclusion:**

This study provides preliminary evidence for the reliability and validity of the Chinese version of the CSI. While the initial results are promising, further research is needed to validate its applicability in various clinical settings and patient populations. The CSI may potentially serve as a useful tool for assessing constipation severity in Chinese patients, but additional validation studies are necessary before its widespread clinical application.

## Introduction

Constipation is a common gastrointestinal dysfunction, with a global prevalence estimated to be between 2 and 35%, depending on variations in population, region, and diagnostic criteria, which significantly impacts patients’ quality of life and healthcare resource utilization (1). The symptoms of this condition are diverse, including difficulty in defecation, reduced frequency of bowel movements, bloating, and abdominal pain (2). Due to the subjectivity and variability of the symptoms, accurately assessing the severity of constipation is crucial for developing personalized treatment plans and evaluating treatment effectiveness (3). In clinical practice and research, the use of standardized assessment tools to quantify the severity of constipation has become an important trend (4).

## Background

In 2008, Professor Madhulika G Varma and her team developed the Constipation Severity Index (CSI) scale (5). The uniqueness of this scale lies in its comprehensive assessment of various aspects of constipation, including difficulties in defecation, abdominal discomfort, and the impact on quality of life (6). The CSI scale consists of 16 items that cover three main dimensions of constipation: defecation, abdominal symptoms, and rectal symptoms. This multidimensional assessment approach makes the CSI a comprehensive and sensitive tool capable of accurately reflecting the severity of constipation in patients (7). Since its introduction, the CSI has been validated and applied in multiple countries and regions, demonstrating good psychometric properties (8, 9). However, despite China being the most populous country in the world and constipation being a common issue among the Chinese population (10), there is currently a lack of a rigorously validated Chinese version of the CSI.

## Purpose

Given the importance of the Constipation Severity Index (CSI) in assessing the severity of constipation and the lack of a validated Chinese version of the scale, this study aims to evaluate the psychometric properties of the Chinese version of the Constipation Severity Index (CSI). Through this research, we hope to provide an effective tool for clinicians and researchers in China to accurately assess and manage patients with constipation (11). Additionally, this study will contribute to the advancement of constipation-related research in China and lay the groundwork for cross-cultural comparative studies (12).

### Data and methods

#### Scale introduction

The Constipation Severity Instrument (CSI) is a validated self-administered scale designed to assess the severity of constipation symptoms, comprising three dimensions with a total of 16 items: obstructive defecation (6 items), colonic inertia (6 items), and pain (4 items). The CSI employs a five-point Likert scale for scoring (0–4 points), with a total score range of 0–73 points; a higher score indicates more severe symptoms. The CSI demonstrates good reliability, with internal consistency (Cronbach’s *α*) ranging from 0.88 to 0.91 and test–retest reliability (intraclass correlation coefficient) ranging from 0.84 to 0.91. The CSI can also distinguish between different types of constipation (such as functional constipation, pelvic floor dysfunction, and constipation-predominant irritable bowel syndrome), with its scores negatively correlated with quality of life. The advantage of the CSI lies in its ability to describe the severity of symptoms and categorize them according to pathophysiological factors.

#### The translation of the scale

To ensure the linguistic validity and accuracy of the Constipation Severity Index (CSI), the research team followed Brislin’s (13) back-translation method and the cross-cultural adaptation guidelines proposed by Beaton et al. (14), conducting a detailed translation-back-translation process. First, two bilingual experts independently translated the original English scale into Chinese, producing T1 and T2 versions. Subsequently, two medical graduate students reviewed and synthesized these versions to form a unified Chinese scale (T version). In the back-translation phase, two bilingual translators, who had not been exposed to the original scale, independently translated the T version back into English, resulting in BT1 and BT2 versions. A master’s student in nursing psychology compared and compiled these two versions to derive the final English back-translation (B version). Then, 11 gastroenterologists and colorectal surgeons were invited for expert review, and the scale was modified based on their feedback. To further ensure the comprehensibility and applicability of the scale, the research team conducted a pre-test study, inviting 30 eligible patients with constipation to participate. This included individuals with varying degrees of constipation and diverse educational backgrounds. Based on the feedback collected, final adjustments were made. Subsequently, the research team verified the translated scale with the original authors to ensure the accuracy of the translation and the preservation of the original meaning. Finally, the translation team collectively compared the original scale, the forward-translated version, and the back-translated version to ensure the linguistic and cultural equivalence of the Chinese version of the CSI while maintaining the content and structural integrity of the original scale. Through this series of rigorous steps, the research team completed the development of the initial Chinese version of the CSI, ensuring that the scale is suitable for Chinese users while retaining its equivalence with the original scale.

#### Pilot survey

We employed a convenience sampling method to select 30 patients suffering from constipation as the subjects of this study at a tertiary referral hospital in Jiangxi Province during May 2024. Inclusion criteria included: age ≥ 18 years and meeting the Rome IV (2) diagnostic criteria for adult functional constipation. Exclusion criteria included constipation caused by opioid medications. All participants completed the initial Chinese version of the Constipation Severity Index (CSI) questionnaire. After the questionnaire was completed, the research team conducted semi-structured interviews with each participant to inquire in detail about the clarity and comprehensibility of each item in the initial Chinese version of the CSI, while recording their feedback and suggestions. Additionally, we collected participants’ basic demographic and clinical characteristics. This pre-survey aimed to evaluate the feasibility and cultural adaptability of the initial Chinese version of the CSI, providing a basis for the subsequent revision of the questionnaire.

#### Expert opinions

To enhance the clarity and comprehensibility of the CSI scale, we consulted 11 experienced surgical experts from various fields, including 4 general surgery specialists and 7 colorectal surgery specialists. The experts reviewed the original CSI and its Chinese draft, assessing the relevance and applicability of the initial Chinese version. This evaluation used a four-point Likert scale, with scores from “not relevant” (1 point) to “highly relevant” (4 points), providing a quantitative assessment of each item’s relevance and appropriateness. After integrating valuable feedback from experts across different fields, we made necessary cultural adjustments to the CSI scale to ensure its content is applicable and accurate within the context of Chinese medical culture. This process has enhanced the scale’s effectiveness and practicality as a tool for assessing constipation severity.

#### Cultural adaptation

The cultural adaptation process of the Severity of Constipation Index (SCI) is a complex and meticulous task that involves adjustments and considerations at multiple levels. Firstly, language expressions have been carefully adjusted while maintaining the integrity of the original structure and content. For example, “Incomplete bowel movements” has been translated as “incomplete bowel evacuation,” which not only accurately conveys the medical meaning but also aligns with Chinese expression habits. The translation of technical terms places particular emphasis on accuracy and comprehensibility, such as “Obstructive defecation subscale” being translated as “obstructive defecation assessment subscale.” Cultural relevance adjustments are another focal point, such as modifying the scoring scale from 0–4 to 1–5, which better fits the cognitive habits of the Chinese audience. When addressing sensitive topics, euphemistic yet precise expressions have been employed to maintain professionalism while considering the attitudes toward such topics in Chinese culture. Simplification and clarification of language are also crucial, as the use of concise and clear Chinese expressions, along with the addition of explanatory phrases (e.g., “for you”), enhances the scale’s approachability and comprehensibility. Furthermore, consistency in the language style of the entire scale has been emphasized, such as standardizing the translation of “bother” to “distress.” During the localization process, expressions familiar to the Chinese audience have been adopted, such as translating “Extremely Severe” contextually as “very severe” or “extremely severe.” To ensure translation quality, it is recommended that both medical and language experts jointly review the work and consider conducting small-scale pre-tests to gather feedback from the target population. Finally, based on feedback from practical applications, continuous optimization of the translation is necessary to ensure the long-term effectiveness of the scale within the Chinese cultural context. This series of meticulous cultural adaptation strategies allows the SCI scale to better integrate into the Chinese cultural context while preserving its original structure and content, significantly enhancing its applicability and credibility among the Chinese population.

#### Demographic of survey population and recruitment

This study used a convenience sampling method to conduct a questionnaire survey from May to September 2024, including two groups: constipation patients and healthy controls. Constipation patients were recruited from the constipation outpatient clinics at two tertiary hospitals in China, where all participants were diagnosed by gastroenterologists according to the Rome IV diagnostic criteria for functional constipation. Inclusion criteria for the patient group included: age ≥ 18 years, meeting Rome IV diagnostic criteria for functional constipation, able to read and understand Chinese, and willing to provide informed consent. Exclusion criteria included: secondary constipation due to organic diseases, opioid-induced constipation, cognitive impairment affecting questionnaire completion, and pregnant or lactating women. For comparison purposes, healthy controls were recruited through online platforms across all provinces in China, with a total of 3,019 healthy participants without constipation symptoms completing the questionnaire online. To ensure the reliability of the Chinese version of the scale, we adhered to measurement research conventions when determining the sample size, which involved multiplying the number of items in the scale by 5–10, while also accounting for approximately 20% of potential invalid questionnaires. Consequently, we initially estimated that the effective sample size should range between 80 and 160, however, considering the statistical requirements for factor analysis, we set the target sample size at 600 (15). Based on this criterion, the research team distributed a total of 650 questionnaires to constipation patients and ultimately retrieved 621 valid responses, resulting in a valid questionnaire rate of 95.5%. This sample size not only meets the basic requirements for scale validation but also provides ample data support for subsequent statistical analyses.

#### Test–retest reliability procedure

To evaluate the temporal stability of the Chinese version of the Constipation Severity Index (CSI), we conducted a test–retest reliability assessment. A subsample of 30 patients who met the inclusion criteria for constipation were selected from the main study population and asked to complete the CSI questionnaire twice, with a two-week interval between the first and second administrations. The two-week interval was selected based on established psychometric literature recommendations (16), as this timeframe is considered optimal for balancing two critical factors: minimizing memory effects where participants might recall their previous responses while ensuring that the underlying construct of constipation severity remains relatively stable, and this interval is widely accepted in scale validation studies for chronic conditions (17). To assess and control for potential memory effects, we implemented the following measures: during the second administration, participants were asked whether they remembered their previous responses, we documented their recall levels to evaluate potential memory bias, and the questionnaires were administered in the same setting and under similar conditions to minimize external influences on responses. For statistical analysis of test–retest reliability, we selected Spearman’s rank correlation coefficient rather than Pearson’s correlation, as preliminary data screening indicated that the CSI scores deviated from normal distribution, and this non-parametric approach is more appropriate for ordinal data and provides a robust measure of consistency between the two time points. A correlation coefficient above 0.70 was considered acceptable, while values above 0.90 were deemed excellent for test–retest reliability (18).

### Statistical methods

All statistical analyses in this study were conducted using R software (version 4.3.2), primarily utilizing the psych package (19) for data processing and preliminary analysis, and the lavaan package (20) for structural equation modeling (SEM) analysis. The study included two samples: one consisting of individuals with constipation (*n* = 621) sourced from hospital patients, and the other comprising a healthy population (*n* = 3,019) recruited voluntarily online from approximately 100 volunteers in each of China’s 30 provinces. To perform comprehensive scale validation, we randomly divided the constipation sample into two groups: one for exploratory factor analysis (EFA, *n* = 310) and the other for confirmatory factor analysis (CFA, *n* = 311). This grouping approach allows us to first explore the latent structure of the scale and then validate this structure through CFA and SEM on an independent sample, thereby enhancing the reliability and validity of the results. CFA, as part of SEM, is used to confirm the factor structure of the scale, assess model fit, and examine the relationships among latent variables. Furthermore, the healthy population sample (*n* = 3,019) will be utilized to conduct known-groups validity analysis, comparing the differences in scale scores between individuals with constipation and healthy individuals, further validating the scale’s discriminative ability.

## Results

**Figure 1:**
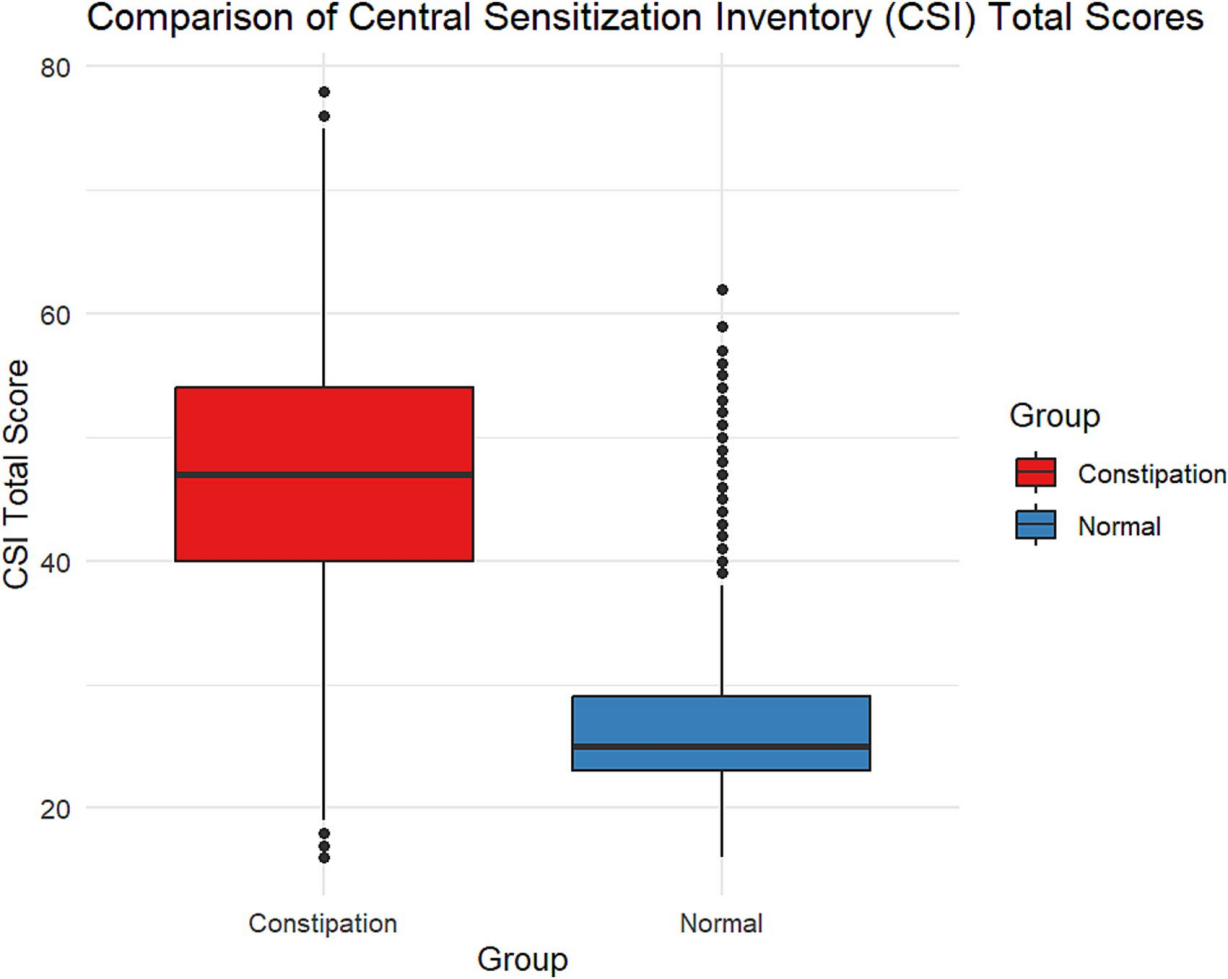
Comparison of central sensitization inventory (CSI) total scores between constipation patients and healthy controls. The box plot shows significantly higher CSI scores in constipation patients (median ≈ 45, range 18–78) compared to healthy controls (median ≈ 28, range 16–62).

This study compared the demographic and clinical characteristics of 621 patients with constipation and 3,019 normal controls, revealing significant differences between the two groups across all study variables (*p* < 0.001). The constipation group had a higher proportion of females (63.1% vs. 52.9%) and an older average age (42.62 years vs. 34.57 years), along with a higher level of education. Regarding lifestyle, the regular exercise rate in the constipation group was significantly lower than that of the normal group (43.6% vs. 73.3%). Clinically, the constipation group displayed a markedly higher laxative usage rate (72.6% vs. 11.7%), and the Constipation Severity Index (CSI) was significantly greater than that of the normal group (median: 47.0 vs. 25.0) as shown in Figure 1. These findings emphasize the significant disparities in demographic characteristics, lifestyle habits, and clinical presentations between patients with constipation and the normal population, providing crucial insights for further understanding and managing constipation. Detailed data can be found in Table 1.

**Table 1:** Comparison of demographic and clinical characteristics between constipation and normal groups.

Variable	Constipation (*n* = 621)	Normal (*n* = 3,019)	*p*-value
Gender			<0.001
Male	229 (36.9%)	1,423 (47.1%)	
Female	392 (63.1%)	1,596 (52.9%)	
age	42.62 (12.50)	34.57 (5.88)	<0.001
Educational level			<0.001
Elementary education or below	20 (3.2%)	626 (20.7%)	
Lower secondary education	35 (5.6%)	1,130 (37.4%)	
Upper secondary education	99 (15.9%)	791 (26.2%)	
Vocational/Technical education	194 (31.2%)	323 (10.7%)	
Bachelor’s degree or equivalent	248 (39.9%)	84 (2.8%)	
Master’s degree or above	25 (4.0%)	65 (2.2%)	
Marital status			<0.001
Married	425 (68.4%)	1790 (59.3%)	
Single	127 (20.5%)	729 (24.1%)	
Divorced/Widowed	69 (11.1%)	500 (16.6%)	
Occupation			<0.001
Employed	385 (62.0%)	1712 (56.7%)	
Unemployed	79 (12.7%)	407 (13.5%)	
Self-employed	110 (17.7%)	899 (29.8%)	
Other	47 (7.6%)	1 (0.0%)	
Yes	0	0	
No	621	3,019	
Exercise			<0.001
Yes	271 (43.6%)	2,212 (73.3%)	
No	350 (56.4%)	807 (26.7%)	
SubstanceUse			<0.001
Yes	159 (25.6%)	897 (29.7%)	
No	462 (74.4%)	2,122(70.3%)	
LaxativeUse			<0.001
Yes	451 (72.6%)	354 (11.7%)	
No	170 (27.4%)	2,665 (88.3%)	
CSI Total median [IQR]	47.0 [40.0, 54.0]	25.00 [23.0, 29.0]	<0.001

SubstanceUse: History of tobacco use or alcohol misuse (yes/no). 2. LaxativeUse: Prior or current use of laxatives or cathartics (yes/no).

### Non-response bias analysis or early-late respondent analysis

To assess potential non-response bias, we conducted early and late evaluations, comparing the top 25% and bottom 25% of respondents on key study variables. The results indicated significant differences between early and late responders in several key characteristics. Late responders had a significantly higher average age (44.36 ± 13.24 years) than early responders (40.83 ± 12.05 years, *p* = 0.015). Regarding occupational scores, early responders (1.87 ± 1.11) scored significantly higher than late responders (1.58 ± 0.92, *p* = 0.011). For exercise habits, early responders (1.64 ± 0.48) were more inclined to engage in physical exercise than late responders (1.48 ± 0.50, *p* = 0.004).

**Figure 2:**
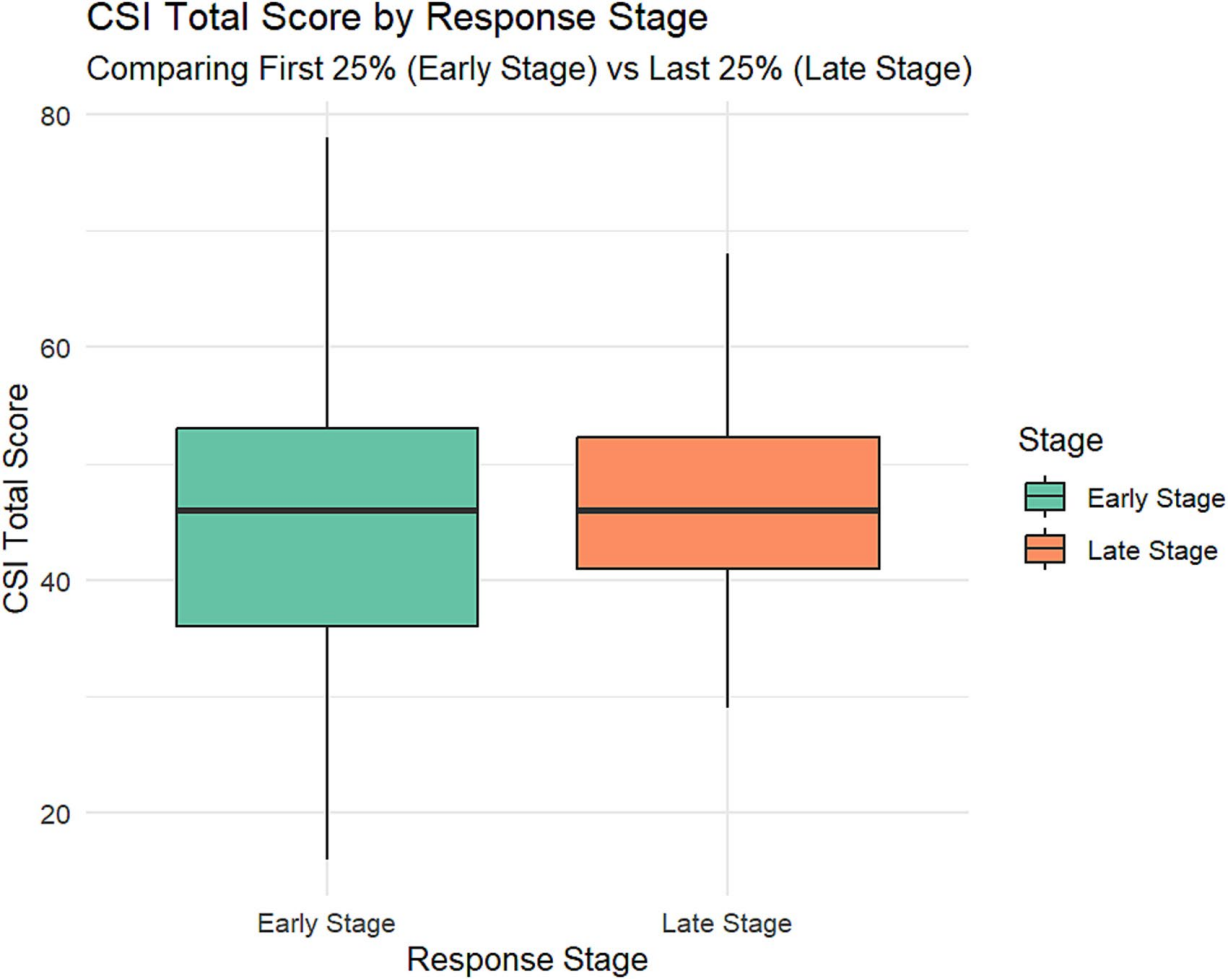
Comparison of central sensitization inventory (CSI) total scores between early-stage and late-stage constipation patients. The box plot displays CSI scores for two groups: early-stage (blue, left) and late-stage (red, right) constipation patients. Late-stage patients exhibit significantly higher CSI scores (median ≈ 50) compared to early-stage patients (median ≈ 40). The late-stage group also shows a broader score distribution, ranging from approximately 25–80, while the early-stage group ranges from about 20–70.

Notably, the total score on the CIS scale showed a difference between early and late responders that was close to but did not reach statistical significance (Early: 44.94 ± 12.30; Late: 47.24 ± 8.58; *p* = 0.057). This result suggests that while there are slight differences, they are insufficient to draw a conclusive association between response timing and CIS scale scores (in Figure 2). This minor discrepancy may be due to sampling error or other unknown factors and requires further validation in a larger sample.

No significant differences were found between early and late responders in other variables such as gender, education level, marital status, substance use, and laxative use (all *p* > 0.05). These findings indicate that although there are differences in certain demographic characteristics and lifestyle habits, early and late responders do not significantly differ on most key variables. Such partial differences may reflect the diversity of the sample rather than systemic non-response bias. However, these differences remain worth considering when interpreting the data and generalizing the results. Detailed data can be found in Table 2.

**Table 2:** Constipation severity index of characteristics between early responders and late responders.

Variable	Early stage (*n* = 156)	Late stage (*n* = 156)	*p*-value
Gender	1.60 (0.49)	1.58 (0.50)	0.731
Age	40.83 (12.05)	44.36 (13.24)	0.015*
Education	4.07 (1.29)	4.27 (0.91)	0.117
Occupation	1.87 (1.11)	1.58 (0.92)	0.011*
Marriage	1.51 (0.71)	1.39 (0.65)	0.116
Exercise	1.64 (0.48)	1.48 (0.50)	0.004*
SubstanceUse	1.71 (0.45)	1.74 (0.44)	0.526
LaxativeUse	1.29 (0.46)	1.22 (0.42)	0.156
TotalScore	44.94 (12.30)	47.24 (8.58)	0.057

Values are presented as mean (standard deviation). * Indicates statistically significant difference (*p* < 0.05).

### Common method bias and discriminant validity assessment

To evaluate potential common method bias and confirm the discriminant validity of the model, we conducted Harman’s single-factor test and heterotrait-monotrait (HTMT) analysis (21, 22). The results of Harman’s single-factor test showed that, based on items 1–16, the proportion of variance explained by the first factor was 40.52%, which is below the critical value of 50%, suggesting that common method bias may not be a significant issue. The HTMT analysis further supported the discriminant validity of the three-factor model, revealing HTMT ratios of 0.771, 0.812, and 0.695 between Factor 1 and Factor 2, Factor 1 and Factor 3, and Factor 2 and Factor 3, respectively, all of which are below the conservative threshold of 0.85. These results confirm the discriminant validity among the factors, indicating not only sufficient differentiation between them but also demonstrating that the measurement model possesses good construct validity, providing statistical support for the three-factor model. These analyses strongly support our measurement model, demonstrating the uniqueness among the constructs and ruling out the significant impact that common method bias may have on the research findings.

### Construct validity

#### Exploratory factor analysis

**Figure 3:**
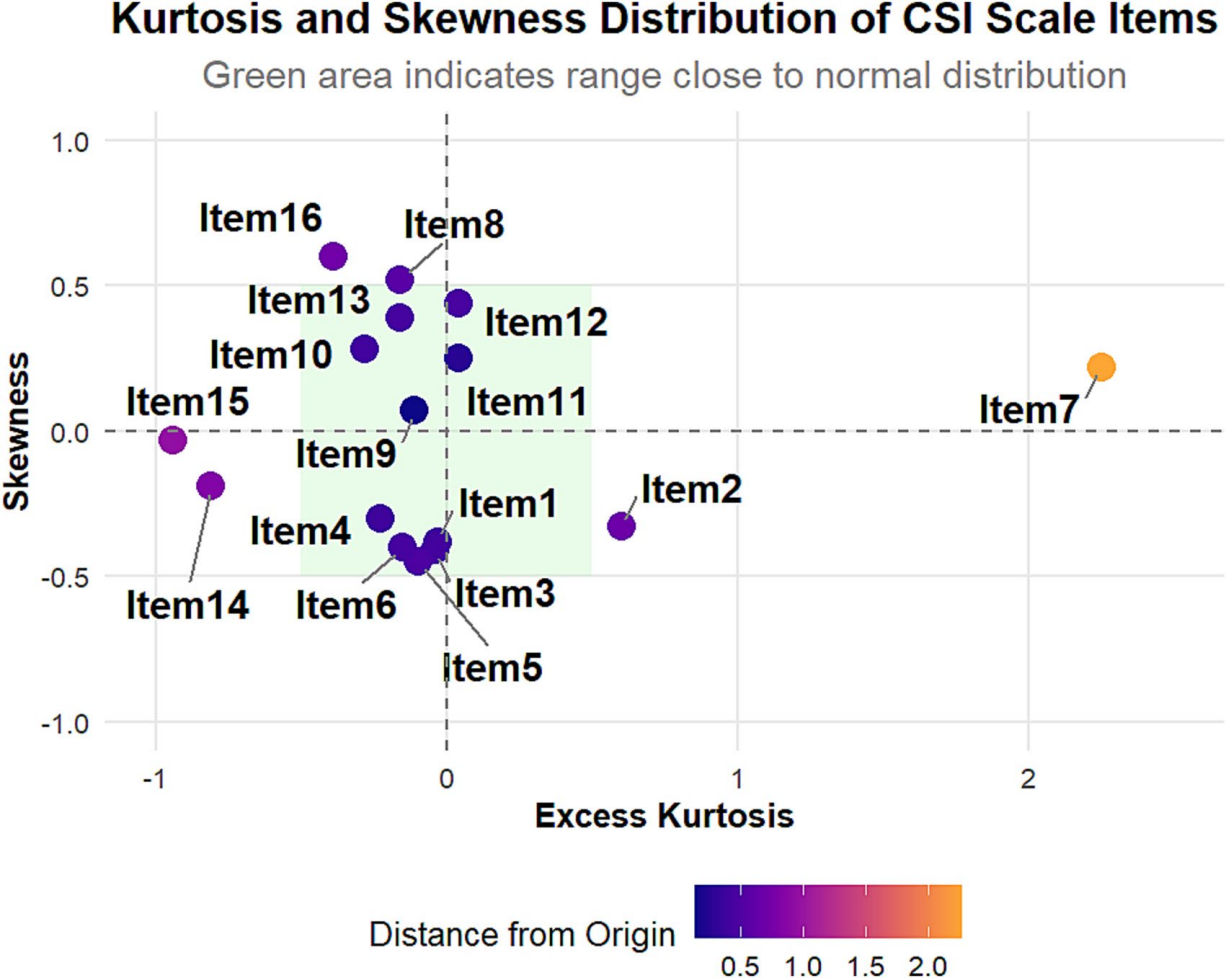
Kurtosis and skewness distribution of central sensitization inventory (CSI) scale items. This scatter plot shows the distribution of excess kurtosis and skewness for the 16 CSI items. Most items (1–6, 8–16) cluster near the center, indicating nearly normal distributions. Item 7 stands out with high positive excess kurtosis and slight positive skewness. Items 8, 12, 13, and 16 display moderate positive skewness, while items 3, 5, and 6 exhibit slight negative skewness. The green shaded area represents the range close to a normal distribution.

To evaluate the structural validity of the scale, we conducted an exploratory factor analysis (EFA). Before performing the EFA, we assessed the normality of the 16 items on the severity of constipation scale. Given the large sample size (*n* > 300), we used the criteria proposed by Kim (2013) (23), which suggest that absolute skewness values greater than 2 or absolute kurtosis values greater than 7 indicate substantively non-normal distributions. The analysis showed that the absolute skewness values for all 16 items were less than 2 (range: 0.03–0.60), and the absolute kurtosis values were also less than 7 (range: 0.03–2.25), indicating that the distributions of all items did not exhibit significant non-normal characteristics. However, we noted some notable distribution features: Items 1 to 6 exhibited a slight negative skew, whereas Items 7 to 16 (excluding Items 14 and 15) showed a slight positive skew; Item 7 had a relatively high kurtosis value (2.25), indicating that the score distribution for this item was somewhat concentrated; Items 14 and 15 displayed relatively low negative kurtosis values (−0.81 and −0.94, respectively), suggesting that the score distributions for these items were relatively flat. Although these features did not violate the assumption of normality, they may reflect differences in participants’ perceptions of various symptoms, warranting further attention in subsequent analyses. Detailed results are presented in Figure 3.

Before conducting exploratory factor analysis, we assessed the data’s suitability. We performed the Kaiser-Meyer-Olkin (KMO) test and Bartlett’s test of sphericity (24). The KMO measure evaluates sample adequacy, with values from 0 to 1. Our analysis yielded a KMO value of 0.947, exceeding the “excellent” threshold of 0.9, indicating the sample is suitable for factor analysis. Bartlett’s test of sphericity assesses whether the correlation matrix significantly differs from an identity matrix, meaning there is significant correlation among the variables. The test results showed a chi-square value (χ^2^) of 9253.41, degrees of freedom (df) = 120, *p* < 0.001. This extremely low *p*-value strongly rejects the null hypothesis (i.e., no correlation among the variables) and further supports the conclusion that the data is suitable for factor analysis.

**Figure 4:**
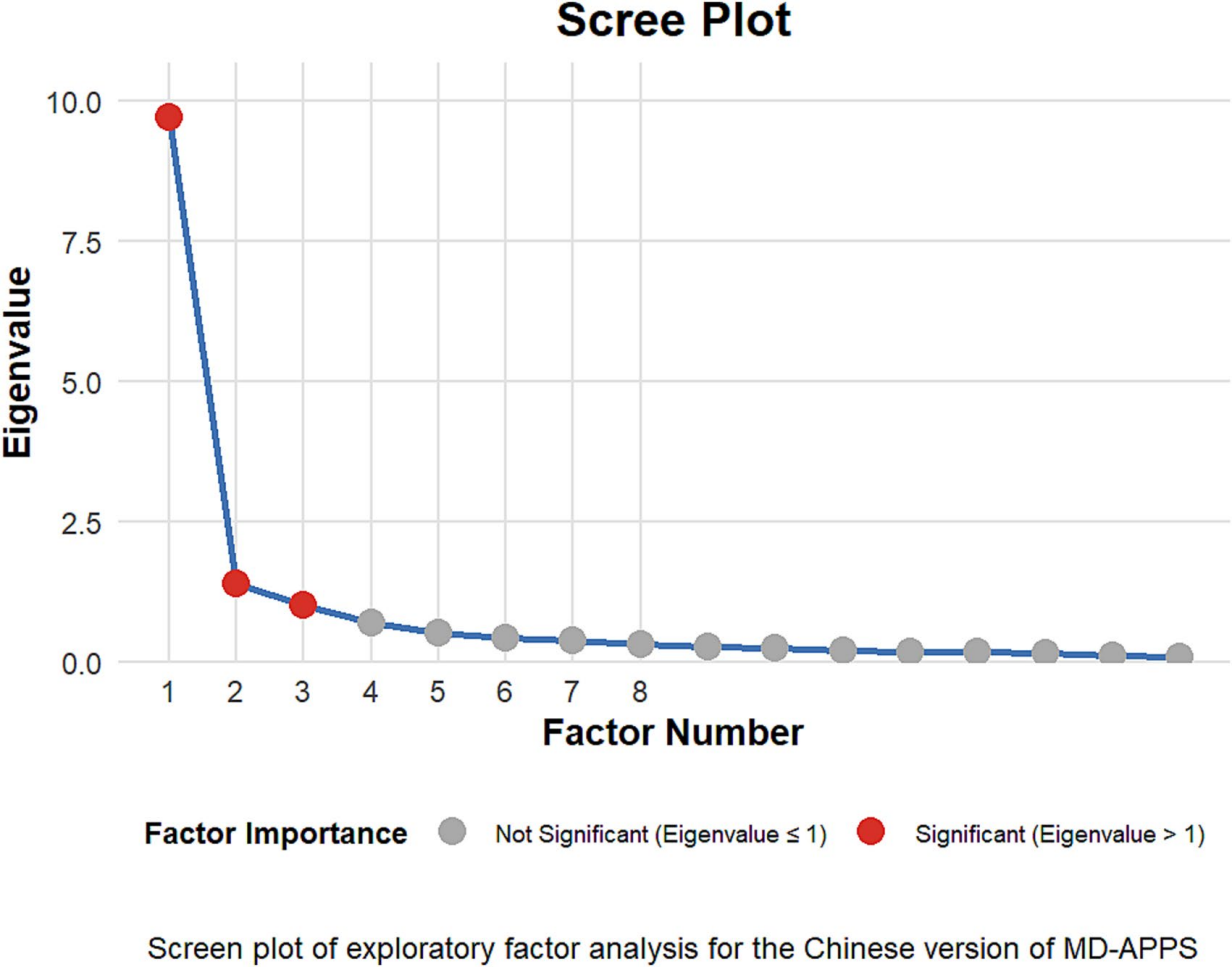
Scree plot of the central sensitization inventory (CSI) scale. This graph illustrates the eigenvalues of factors extracted from the CSI scale. The plot shows a distinct “elbow” or point of inflection after the third factor, suggesting a three-factor structure for the CSI. The first three factors have eigenvalues above 1, with a sharp decline in eigenvalues after the third factor. This three-factor solution indicates that the CSI scale can be effectively represented by three main underlying dimensions or constructs, which may correspond to different aspects of central sensitization symptoms or experiences.

The exploratory factor analysis results show that the Chinese version of the CSI scale has a three-factor structure (ML3, ML1, and ML2) (in Figure 4), accounting for 71.81% of the total variance. These factors explain 28.92, 23.09, and 19.81% of the variance, respectively. Factor ML3 includes Items 1 to 6, with factor loadings from 0.75 to 0.85; ML1 includes Items 13 to 16, with factor loadings from 0.86 to 0.93; and ML2 includes Items 7 to 12, with factor loadings from 0.45 to 0.95. The communalities (h2) of all items exceed 0.40, and the corrected item-total correlations are above 0.50, indicating each item’s strong contribution to its factor. Notably, Items 7 and 11 in factor ML2 have relatively low factor loadings (0.45 and 0.46, respectively), which may require further attention. Detailed data is available in Table 3.

**Table 3:** Factor analysis and total correlation of CSI Chinese version (*N* = 621).

Item *N* = 310	Factor loading	h2	U2	Corrected items-total correlation	Cronbach’s alpha	McDonald’s Omega	% Of the variance explained
ML3					0.929	0.930	28.92
Item1	0.75	0.74	0.26	0.79			
Item2	0.85	0.62	0.38	0.68			
Item3	0.82	0.75	0.25	0.79			
Item4	0.77	0.68	0.32	0.76			
Item5	0.79	0.73	0.27	0.78			
Item6	0.77	0.71	0.29	0.77			
ML1					0.902	0.904	23.09
Item13	0.93	0.85	0.15	0.79			
Item14	0.90	0.92	0.08	0.83			
Item15	0.88	0.90	0.10	0.83			
Item16	0.86	0.72	0.28	0.72			
ML2					0.931	0.933	19.81
Item7	0.45	0.41	0.59	0.54			
Item8	0.95	0.83	0.17	0.67			
Item9	0.66	0.67	0.33	0.71			
Item10	0.79	0.74	0.26	0.72			
Item11	0.46	0.60	0.40	0.72			
Item12	0.54	0.63	0.37	0.74			
Total					0.936	0.937	71.81

#### Confirmatory factor analysis

**Figure 5:**
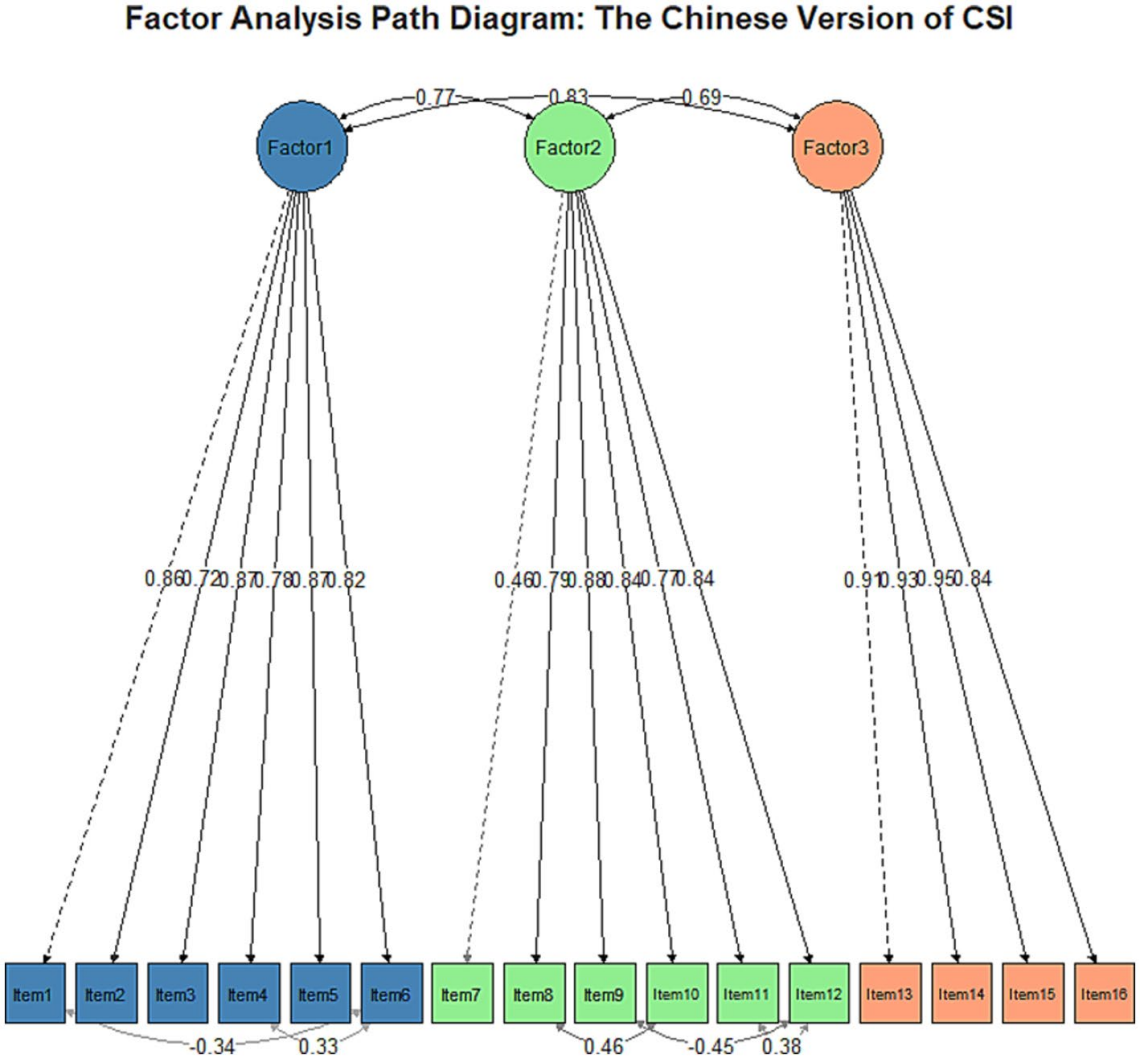
The confirmatory factor analysis (CFA) path diagram for the Chinese version of the Central Sensitization Inventory (CSI) supports a three-factor structure. Factor 1 (items 1–6), Factor 2 (items 7–11), and Factor 3 (items 12–16) exhibit strong inter-factor correlations (0.69–0.83) and generally high factor loadings (0.46–0.84). Most items load well onto their respective factors, with some cross-loadings observed. This structure suggests that the Chinese CSI effectively measures three distinct but related dimensions of central sensitization, providing evidence for its construct validity in Chinese populations and supporting its use as a multidimensional assessment tool in clinical and research settings.

The results of the confirmatory factor analysis support the three-factor structure of the Chinese version of the CSI, showing a good model fit: χ^2^/df = 2.68 [<3 indicates good (25)], CFI = 0.965, and TLI = 0.956 (both > 0.95 indicate good), RMSEA = 0.074 [90% CI: 0.063–0.085] (< 0.08 indicates acceptable), and SRMR = 0.047 (< 0.08 indicates good) (26). The standardized factor loadings for all items exceed 0.40 (> 0.50 indicates acceptable), specifically ranging from: Factor 1 (0.724–0.873), Factor 2 (0.462–0.882), Factor 3 (0.840–0.946) (see Figure 5). The inter-factor correlations are moderate (0.687–0.835, < 0.85 indicates that the factors have discriminant validity), indicating that the scale has good construct validity.

#### Reliability analysis

The reliability of the Chinese version of the CSI was assessed using multiple methods to thoroughly evaluate the internal consistency and item quality of the scale. The results showed that the overall internal consistency of the Chinese version of the CSI is strong, with a Cronbach’s *α* of 0.956, a standardized Cronbach’s α of 0.956, and a McDonald’s *ω* of 0.937. The reliability of each factor was also high: the Cronbach’s α for the ML3 factor (Factor 1) was 0.929, and McDonald’s ω was 0.930; for the ML1 factor (Factor 2), Cronbach’s α was 0.902, and McDonald’s ω was 0.904; for the ML2 factor (Factor 3), Cronbach’s α was 0.931, and McDonald’s ω was 0.933.

The item analysis results supported the scale’s quality. The corrected item-total correlations for all items ranged from 0.53 to 0.83, surpassing the commonly recommended threshold of 0.3. Notably, Item 15 (*r* = 0.83) and Item 14 (*r* = 0.83) demonstrated strong correlations, suggesting they may be among the most representative items on the scale; conversely, Item 7 (*r* = 0.54) had a relatively lower correlation but remained within an acceptable range, warranting further attention. The average inter-item correlation was 0.58, indicating a certain degree of association among the item.

#### Content validity

Based on evaluations by 11 experts, the Item-Level Content Validity Index (I-CVI) of the CSI ranges from 0.85 to 1.0, indicating that each item has good content relevance. Meanwhile, the Scale-Level Content Validity Index (S-CVI/UA) reaches 0.9, reflecting the experts’ recognition of the scale’s overall content. These results suggest that the Chinese version of the CSI has maintained good content validity during the cultural adaptation process.

#### Test–retest reliability

The test–retest reliability analysis demonstrated good temporal stability of the Chinese version of the CSI. Spearman correlation coefficients for individual items ranged from 0.912 to 0.972 (all *p* < 0.05), with an overall average consistency coefficient of 0.953. When participants were asked about their recall of previous responses during the retest, most reported minimal to no memory of their exact answers, indicating negligible memory effects on the reliability results. These findings suggest that the Chinese version of the CSI maintains satisfactory stability over a two-week interval, supporting its use for repeated measurements in both clinical practice and research settings.

## Discussion

The research revealed significant differences in demographic characteristics, lifestyle, and clinical manifestations between patients with constipation and a normal control group, providing important insights for a deeper understanding and management of constipation. The higher proportion of females and older average age in the constipation group may be related to hormonal changes, lifestyle factors, and other elements, which is consistent with previous research findings (33). Notably, the higher education level in the constipation group may influence their health awareness and healthcare-seeking behavior, which deserves further exploration. The significantly lower rate of physical activity in the constipation group (43.6% vs. 73.3%) underscores the importance of lifestyle interventions in managing constipation, aligning with existing literature that emphasizes the positive impact of exercise on intestinal function (27). The laxative usage rate in the constipation group reached 72.6%, raising concerns about drug dependence and potential side effects, underscoring the need for safer and more effective strategies for managing constipation (28). The significant difference in CSI scores (median: 47.0 vs. 25.0) not only validates the discriminative ability of this scale but also supports its clinical application as a tool for assessing constipation severity.

Regarding methodology, the analysis of non-response bias reveals that early and late respondents differ in certain characteristics, such as age and exercise habits, suggesting the need to consider sample diversity when interpreting results (29). However, most key variables show no significant differences, enhancing the study’s reliability. Analyses of common method bias and discriminant validity further support the construct validity of the Chinese version of the CSI, with results from the Harman single-factor test and HTMT analysis indicating that the scale possesses good psychometric properties.

Exploratory factor analysis (EFA) identified a three-factor structure accounting for 71.81% of the total variance, indicating the scale effectively captures multiple dimensions of constipation symptoms. Confirmatory factor analysis (CFA) further validated this structure, demonstrating a good model fit (χ^2^/df = 2.68, CFI = 0.965, TLI = 0.956, RMSEA = 0.074, SRMR = 0.047), conforming to recognized psychometric standards. This result not only validates the structure of the original English version of the Constipation Symptom Inventory (CSI) but also suggests the scale maintains good construct validity within the Chinese cultural context.

Reliability analysis showed that the Chinese version of the CSI demonstrates excellent internal consistency (Cronbach’s *α* = 0.956, McDonald’s *ω* = 0.937), with each factor’s reliability exceeding 0.90. This finding aligns with the high reliability of the original scale, indicating the Chinese version of the CSI possesses a high degree of reliability in measuring the severity of constipation symptoms. Item analysis revealed that the corrected item-total correlation coefficients for all items exceeded 0.50, further confirming the scale’s internal consistency and item quality.

The results of the content validity analysis (I-CVI range 0.85–1.0, S-CVI/UA = 0.9) indicate that the expert panel highly recognized the relevance and representativeness of the scale’s content. This reflects not only the quality of the translation process but also demonstrates that the Chinese version of the CSI has maintained good content validity during the cultural adaptation process. According to the standards set by Polit and Beck (2006), an I-CVI greater than 0.78 and an S-CVI/UA greater than 0.8 are considered to have excellent content validity; our results clearly exceed this standard (30). This finding is consistent with the results of other successful cross-cultural adaptation studies, such as those emphasized by Sousa and Rojjanasrirat (2011), which highlight that a high-quality translation and cultural adaptation process are crucial for maintaining the content validity of the scale (31).

The reliability analysis of test–retest conducted at two-week intervals (Spearman coefficient range 0.912–0.972) confirms the scale’s stability, supporting its use in clinical follow-up and longitudinal studies. This result aligns with the excellent test–retest reliability standards proposed by Koo and Li (2016), suggesting that an ICC (or equivalent metric in non-parametric cases) greater than 0.90 indicates outstanding scale stability (18).

It is noteworthy that, although most items performed well, items 7 and 11 in the ML2 factor exhibited relatively low factor loadings (0.45 and 0.46, respectively). This may reflect the particularities of these items within the Chinese cultural context or suggest potential differences in the manifestation of constipation symptoms (14). Additionally, the high kurtosis value for item 7 (2.25) and the low negative kurtosis values for items 14 and 15 may reflect unique perception patterns among patients regarding different constipation symptoms (32), providing interesting directions for future research.

In summary, these findings indicate that the Chinese version of the CSI is an effective, reliable, and culturally adapted tool suitable for assessing the severity of symptoms in Chinese patients with constipation. It offers a valuable assessment tool for clinical practice and provides a reliable measurement instrument for constipation-related research.

## Limitations

Despite achieving positive results in validating the Chinese version of the CSI, this study has notable limitations. First, although the sample size is relatively large, it may not fully represent all regions and cultural backgrounds of patients with constipation, potentially limiting the generalizability of the findings. Our convenience sampling from tertiary hospitals may introduce selection bias, as our current sample might overrepresent patients with more severe symptoms and underrepresent those with milder conditions or those managed in primary care settings. Second, items 7 and 11 in the ML2 factor show relatively low factor loadings (0.45 and 0.46), which might affect the internal consistency of the scale. Although the test–retest reliability analysis demonstrated good stability, the small sample size (*n* = 30) and the two-week interval may not be sufficient to assess long-term stability. Furthermore, this study lacks a comprehensive evaluation of criterion-related validity and predictive validity. Notably, some items (e.g., items 7, 14, and 15) exhibited unique distribution characteristics, warranting further exploration of the underlying reasons. Given these limitations, future research should consider conducting large-sample, multi-center longitudinal studies to explore the integration of the CSI with objective physiological indicators and further assess its applicability in different populations and clinical settings. In future validation studies, we plan to employ stratified sampling across various healthcare settings (including community clinics and secondary hospitals) to ensure better representation of the diverse constipation patient population in China, which would enhance the overall psychometric properties and clinical value of the scale.

## Conclusion

The Chinese version of the CSI has undergone rigorous psychometric evaluation, demonstrating strong reliability and validity. Both exploratory and confirmatory factor analyses support a three-factor structure, explaining a significant portion of the total variance, with good model fit indices. The scale shows strong internal consistency, good content validity, and stable test–retest reliability. Collectively, these results indicate that the Chinese version of the CSI is a reliable, effective, and stable tool suitable for assessing the severity of symptoms in constipation patients within a Chinese-speaking context, providing a valuable measurement instrument for clinical practice and research.

### Statement on scale usage and authorization

The Constipation Severity Instrument (CSI) used in this study was originally developed by Professor Madhulika G. Varma, MD. The scale has been translated into Chinese and is cited appropriately in this paper. We have obtained explicit permission from Professor Varma to use and translate the scale for our research.

The original scale was published in Varma et al. (5).

The Chinese version of the CSI used in this study has undergone rigorous translation and validation processes to ensure its reliability and validity in the Chinese context. All rights to the original English version of the CSI remain with the original authors.

## Data Availability

The raw data supporting the conclusions of this article will be made available by the authors, without undue reservation.
